# Diffusion and upscaling of municipal climate mitigation and adaptation strategies in Germany

**DOI:** 10.1007/s10113-022-02020-z

**Published:** 2023-01-20

**Authors:** Kristine Kern, Peter Eckersley, Wolfgang Haupt

**Affiliations:** 1 Research Group Urban Sustainability Transformations, Leibniz-Institut für Raumbezogene Sozialforschung IRS eV, Erkner, Germany; 2 Faculty of Social Sciences, Business and Economics, Ảbo Akademi University, Turku, Finland; 3 Nottingham Business School, Nottingham Trent University, Nottingham, UK

**Keywords:** Policy diffusion, Policy upscaling, Municipalities, Local climate mitigation, Local climate adaptation, Germany

## Abstract

Drawing on data for the 104 largest German cities, and deeper analysis of six mid-sized cities (including forerunners, followers and latecomers in climate mitigation and adaptation), we find that the spread of local mitigation and adaptation strategies across Germany can be explained by a combination of horizontal diffusion and vertical upscaling. Specifically, while the spread of climate mitigation initiatives in the 1990s was triggered primarily by transnational municipal networks (horizontal diffusion), the development and revision of climate mitigation strategies and the emergence of climate adaptation strategies during the last decade have been driven mainly by national and subnational funding programmes (vertical upscaling). Notably, forerunner cities are less dependent on external funding than followers and latecomers, because they have more internal capacity to act. By arguing that upscaling of local climate policies from forerunners to followers and latecomers depends on interventions by national and subnational authorities, we stress that the majority of German municipalities require external support in order to develop and implement effective climate strategies.

**Supplementary Information:**

The online version contains supplementary material available at 10.1007/s10113-022-02020-z.

## Introduction

Since the early 1990s, an increasing number of cities across the world have adopted plans and strategies for climate mitigation and adaptation. Such documents set out the measures they intend to adopt to tackle climate challenges within the local context. Analyses of mitigation strategies have examined and compared their contents, focusing for example on the extent to which they might achieve CO_2_ emission reductions or carbon neutrality (Salvia et al 2021). Given that the true scale and exact timing of climate change impacts are extremely difficult to predict, comparisons of adaptation strategies have tended to identify the risks that they seek to combat (e.g., heatwaves or flooding) and/or the sectors in which they propose taking action (Otto et al 2021).

There is a substantial literature on the reasons why many municipalities have become engaged in climate policy (e.g., Bulkeley 2010; Howarth et al 2022). Studies have also examined the factors that shape climate policymaking within cities, particularly in the Global North (Eckersley 2018; Kern 2019; Schulze and Schoenefeld 2022). However, despite a general understanding that codifying proposed initiatives in a plan or strategy greatly increases the likelihood that they will be implemented (Heidrich et al 2017), research has focused on large forerunner cities. We know much less about how followers and latecomers may be adopting the approaches of these forerunners within their own municipal contexts (Haupt and Kern 2022).

In Germany, the first municipal mitigation strategies appeared and spread in the 1990s, whereas adaptation plans and strategies have emerged and spread during the last decade. This paper develops a conceptual approach to explain how their spread has been influenced by horizontal and vertical forms of policy diffusion and upscaling. By combining data for the 104 largest German cities with analysis of six mid-sized cities (including forerunners, followers and latecomers in mitigation and adaptation), we identify patterns in their adoption of climate strategies, and analyse the key drivers of local action.

## Diffusion and upscaling of policy innovations

The concept of policy diffusion can help to explain the rapid spread of climate strategies across cities. This perspective first took hold in the 1960s with studies of how different American states adopted similar approaches (Karch 2007; Kern 2000; Gray 1973; Walker 1969), and has since been applied to many policy areas, including climate and energy policies (Kammerer and Namhata 2018; Schoenefeld et al. 2022). Rogers (2003, p. 5) defines diffusion as “the process by which an innovation is communicated through certain channels over time amongst the members of a social system.” Thus, policy diffusion can be characterised by three factors: (1) policy innovations; (2) channels of diffusion, and (3) adopting organisations.

Policy innovations are new and innovative programmes, rules and practices. Comparative studies focus in particular on the adoption of new legislation. In the area of climate and energy policy, this includes for example climate change acts and renewable energy mandates. Communication channels, such as cross-border networks and intergovernmental institutions, shape the cumulative adoptions of an innovation over time. Transfer agencies, i.e. institutionalised forms of cooperation and coordination such as Germany’s Centre for Climate Adaptation (https://zentrum-klimaanpassung.de/), can influence the speed of diffusion. These factors are essential for the diffusion lifespan of a policy innovation, which encompasses several stages (innovation, early adoption, early majority, late majority and laggard) through which a typical innovation passes when it spreads (Mallinson 2021). Adopting organisations such as states or cities differ considerably with respect to resources and capacities, which contribute towards them becoming leaders or laggards in diffusion processes. Relevant factors include the size and wealth of a municipality, absence of carbon-intensive industries, strength of the Green Party, engagement of civil society and presence of higher education and research organisations (Haupt et al 2022; Schulze and Schoenefeld (2022); Abel 2021). As with other theories of policy change, both internal factors (such as political preferences) and external conditions (such as city networks) can shape how policy innovations diffuse across different jurisdictions. Diffusion patterns can be influenced by external events that focus attention: for example, previous research has shown that it can occur rapidly following international conferences (Karch et al. 2016; Strebel 2011; Kern 2000).

Diffusion studies tend to focus on horizontal dynamics between organisations that operate at the same level (such as nation-states, states in a federal system or municipalities). Research into vertical diffusion is more limited (Shipan and Volden 2012), and those studies that do exist tend to examine how ideas and policies travel in a “bottom-up” rather than “top-down” direction. Examples include the vertical diffusion of anti-smoking policies from US cities to states (Shipan and Volden 2006) or the diffusion of climate change acts from German states (*Länder*) to the federal government (Eckersley et al 2021).^1^ In practice, we often find combinations of horizontal and vertical diffusion because the former may eventually lead to the latter and vice versa.

Four mechanisms have become dominant in the discussion on policy diffusion: coercion, competition, emulation and learning. However, in line with other scholars (Kuhlmann 2021; Maggetti and Gilardi 2016), we only consider voluntary forms of diffusion and therefore exclude coercion which is by definition not voluntary. Instead, we combine voluntary diffusion with upscaling approaches (Kern 2019; van Doren et al 2018; van Winden and van den Buuse 2017). The World Bank defines upscaling as “expanding, adapting and sustaining successful policies, programmes or projects in different places and over time to reach a greater number of people” (World Bank 2005). In contrast to voluntary policy diffusion, upscaling requires a certain degree of state intervention, i.e. involvement of higher levels of government. There is some overlap between diffusion and upscaling, but in this paper we limit upscaling to “soft” governmental interventions, ranging from coordination (such as the coordination of networks between municipalities) to guidelines and subsidies provided by states and the federal government (Kern 2019).

As climate policy is still a voluntary task of German municipalities, we distinguish between horizontal and vertical upscaling: Horizontal upscaling resembles horizontal diffusion, but it highlights the involvement of governmental actors at higher levels of government. It aims for the exchange of experiences, knowledge transfer and learning amongst forerunners. An example would be a network for municipal climate managers initiated by the federal government, which was set up to facilitate the exchange of experience between them. Vertical upscaling refers to interdependent relations and combinations of bottom-up and top-down processes. It ranges from the development of new institutions such as regional energy agencies to project funding by the federal government.

Based on this approach, our analysis includes four dimensions, which explain the spread of climate strategies amongst municipalities: (1) horizontal diffusion; (2) vertical diffusion; (3) horizontal upscaling; and (4) vertical upscaling. Table 1 sets out the four dimensions of diffusion and upscaling, and illustrates them with examples.

**Table 1:** Dimensions of diffusion and upscaling of policy innovations

Dimensions	Characteristics	Examples
Horizontal diffusion	Voluntary adoption of similar policies at the same level of government	Membership of transnational municipal networks; voluntary spread of local experiences within these networks from forerunners to followers and latecomers
Vertical diffusion	Voluntary adoption of similar policies across different levels of government	Introduction of a federal funding scheme for municipalities, based on the experiences of state governments
Horizontal upscaling	Initiatives of higher levels of government that facilitate the adoption of similar policies at the lower level by horizontal interaction at the same level of government	Federal or state governments as moderators of horizontal networks amongst forerunner cities
Vertical upscaling	Initiatives at higher levels of government that facilitate the adoption of similar policies at the lower level by vertical interaction across different levels of government	Federal or state subsidy schemes for all municipalities so that followers and latecomers can keep pace with forerunners

We anticipate that structural, socioeconomic and political conditions within individual municipalities shape the adoption of policy innovations and the nature of policy diffusion and upscaling. Specifically, larger, wealthier municipalities that are more reliant on service industries, and which have growing, younger populations, universities and/or research institutions, and strong Green Party and civil society representations, may join and be more active in climate networks and therefore adopt climate strategies by voluntary diffusion. Conversely, municipalities in which less favourable conditions apply will probably rely more on upscaling and initiatives by higher levels of government (in particular federal and state-funded subsidy schemes).

## Methods

We study the case of Germany, which is a fairly decentralised federal state and widely considered a national climate policy leader. Germany represents a highly relevant country to study local climate policy diffusion and upscaling, given that many cities developed climate mitigation strategies from the 1990s onwards and have supplemented them with climate adaptation strategies during the last 10 years. Previous studies of climate plans and strategies in Germany and elsewhere have highlighted the importance of these documents for setting priorities, mobilising local resources and effecting policy change (Salvia et al 2021; King 2022). As climate change is still a voluntary activity for municipalities, any strategy that they adopt would not be the result of coercion by the federal or state-level.

We selected six case-study cities drawing on the ranking developed by Otto et al (2021), which examined the 104 largest German cities^2^ and addressed mitigation and adaption separately. In addition, we drew on the project funding information system (*profi-Datenbank*) of the German federal government’s funding programme *Kommunalrichtlinie* (*KRL*) to identify the spread of climate plans and strategies across German cities (see also BMU 2021). We also analysed membership data of the Climate Alliance, in particular the date of entry into this transnational municipal network (Klimabündnis 2022). On this basis, we examined mitigation and adaptation strategies in six cities, which are located in four federal states in different parts of the country (Table 2). In three of these cities, we focused more on climate mitigation and in the remaining three we concentrated on climate adaptation. Since we were also keen to examine cities that adopted climate strategies at different points in time, we selected one “forerunner” city, one “follower” city and one “latecomer” city for each area. The forerunners (Aachen, Karlsruhe) belong to the small group of local governments that have pioneered climate strategies in Germany. Followers (Remscheid, Oberhausen) became active earlier than most German municipalities but were able to refer to the examples of the forerunners. Latecomers (Würzburg, Brandenburg/Havel) started their activities later than most other cities (see Table 2).

**Table 2:** General characteristics of case-study cities

City (state)	City type	Population	GDP per capita in €^1^	Political situation since 1990^2^
Aachen (AA) (North Rhine-Westphalia)	Mitigation forerunner	248,878	39,194	Mayors: switch between CDU and SPD, Green mayor since 2020 City Council: continuous CDU dominance
Remscheid (RS) (North Rhine-Westphalia)	Mitigation follower	113,849	37,671	Mayors: mostly SPD mayors City Council: continuous SPD dominance
Würzburg (WÜ) (Bavaria)	Mitigation latecomer	126,954	67,017	Mayors: switch between CSU, SPD and independent, Green Deputy Mayor since 2020 City Council: continuous CSU dominance, Greens strongest party since 2020
Karlsruhe (KA) (Baden-Württemberg)	Adaptation forerunner	308,436	66,579	Mayors: switch between CDU and SPD City Council: CDU dominance, Greens strongest party since 2020
Oberhausen (OB) (North Rhine-Westphalia)	Adaptation follower	209,566	27,489	Mayors: mostly SPD, CDU mayor since 2015 City Council: continuous SPD dominance, CDU strongest party since 2020
Brandenburg an der Havel (BB) (Brandenburg)	Adaptation latecomer	72,040	33,053	Mayors: SPD until 2003, CDU since then City Council: continuous SPD dominance until 2003, CDU dominance since 2003

^1^Figures for the city region Aachen (556,246 inhabitants)

^2^*CDU* Christian Democratic Party, *CSU* Christian Social Union, *SPD* Social Democratic Party

We chose mid-sized cities because in this category we find the best mix of forerunners, followers and latecomers: cities above 500,000 inhabitants are almost all forerunners, and the majority of smaller cities belongs to the group of latecomers and laggards. In total, we conducted fifteen interviews between 2018 and 2021 across the six cities (see annex). Due to the Covid-19 pandemic, we undertook this fieldwork either by video conference or by telephone. Given that most mid-sized municipalities only employ a small number of staff on climate issues, there was a limited number of people we could speak to in each of our cities. Our interviewees were keen to retain confidentiality, and therefore we have not made the transcripts of our interviews publicly available. We also analysed key strategic documents (e.g. mitigation and adaption strategies) produced by each city, and consulted the websites of municipal climate networks and state and federal government agencies in Germany that provide funding for local climate action.

## Diffusion and upscaling of climate policies

### Driving factors for diffusion and upscaling of climate policies

Since the late 1980s, a range of factors have contributed towards German cities acting to tackle climate change, initially in terms of mitigation and subsequently in the area of adaptation. Apart from internal drivers (such as a city’s size and wealth), local climate action was driven by external factors. Triggered by the Chernobyl disaster, Freiburg introduced its first energy concept (*Energieversorgungskonzept*) in 1986. Cities such as Munich, Hamburg and Berlin soon followed with similar initiatives.

Transnational municipal networks, in particular the Climate Alliance (*Klimabündnis*), facilitated climate policy diffusion. Like other networks in this policy area, the Climate Alliance emerged shortly before the Rio Conference in 1992 (Kern and Bulkeley 2009). Founded in 1990, it has been the largest of these networks from the outset. By March 2022, it had more than 1800 full members in 28 European countries, including almost 600 members in Germany. When the Climate Alliance was created, member cities committed voluntarily to reduce CO_2_ emissions by 50% by 2010 (compared to 1990 levels). To join the network, a city council had to pass a formal decision to endorse this target, which led to many members developing mitigation strategies. However, as even the most progressive cities could not reach this ambitious goal, it was lowered in 2006. Since then, members commit themselves to (1) a continuous reduction in CO_2_ emissions by 10% every 5 years, (2) halving of per capita CO_2_ emissions by 2030, and (3) a long-term target of 2.5 t CO_2_ emissions per capita annually (Climate Alliance 2021).

**Fig. 1:**
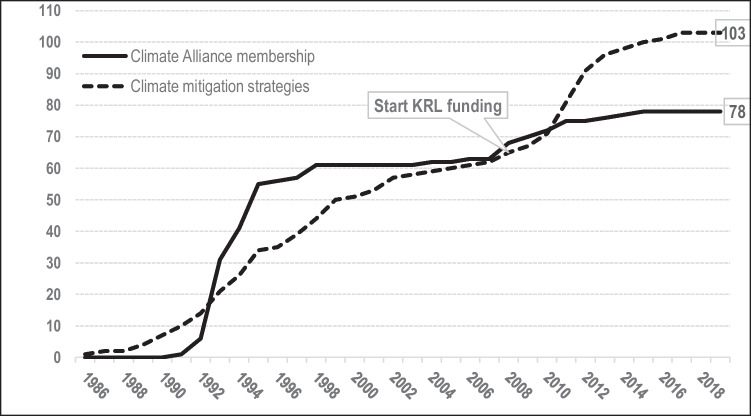
Climate Alliance membership and climate mitigation strategies among the 104 largest German cities (KRL: Kommunalrichtlinie)

Figure 1 tracks the cumulative number of climate mitigation strategies adopted by the largest 104 German cities (Otto et al 2021). It shows how the initial spread of strategies coincides with the rapid diffusion of Climate Alliance membership. Forty-one cities joined the network in the early and mid-1990s and developed their first energy and climate plans before the turn of the century; notably, 31 of them had a strategy in place by 1995 (including Aachen and Remscheid). Twelve other cities (including Karlsruhe) also adopted a strategy or plan before 2000 but did not join the Climate Alliance in this early phase: only five of these municipalities had set up a strategy by 1995. Another 20 cities (including Oberhausen) joined the Climate Alliance in the early and mid-1990s, but became late adopters due to having fewer capacities (smaller size, lack of financial resources, etc.). In addition, almost all of the 31 latecomers (including Würzburg and Brandenburg/Havel) set up their climate strategies after 2008. Figure 1 illustrates how the adoption of climate mitigation plans and strategies followed a classic S-curve of horizontal diffusion until the mid-2000s, in that neither state nor federal governments influenced municipalities to develop these documents. Notably, however, rapid diffusion of Climate Alliance membership had stopped by the late-1990s.

Prior to 2008, funding of local climate policy was restricted to a few German states*.* In order to provide support for municipalities where state funding streams were lacking, the federal government stepped in and set up the KRL scheme, which took the experiences at state level into account. The example of the state of Baden-Württemberg provides evidence for vertical diffusion. Members of the state parliament (*Landtag*) from Freiburg lobbied at state level to set up the *Klimaschutz-Plus* scheme in 2002, which included funding for municipalities. As part of this programme, the state has paid 25% instead of 20% of a municipality’s total costs in cases where it also receives KRL funding (Graf et al 2018). Coupling the state and federal funding programmes helped to stimulate the introduction of local climate strategies in Baden-Württemberg.

Between 2008 and the end of 2021, the KRL programme supported around 21,500 projects in more than 4450 German municipalities (i.e. more than 40% of all municipalities) with around 965 million Euros (Nationale Klimaschutzinitiative 2022). While all but one of the 190 cities and towns with more than 50,000 inhabitants have applied for funding, smaller municipalities are more difficult to reach (BMU 2021). Although KRL funding is not competitive and guidance on how to fulfil the requirements is available, municipalities need certain capacities to apply (see also Zeigermann et al. 2022).

The KRL programme was extended various times to cover more eligible applicants and areas of funding. For example, it has financed infrastructure projects such as investments in energy-efficient street lighting, alongside municipal climate strategies and energy management. Thus, the programme became an essential driver for the development of local climate policies. Mid-sized and small municipalities would not have had sufficient resources to employ staff and develop strategies without this support (BMU 2021). Indeed, between 2008 and 2019, the scheme supported over 1300 initiatives to develop climate strategies, including around 700 integrated mitigation and adaptation strategies (BMU 2021). As municipalities have shifted their attention to implementation (Otto et al 2021), the number of applications to fund climate managers (who are recruited primarily to prepare and implement strategies) increased during the same period. The KRL scheme covers the costs for such positions for an initial period of 3 years, with the option to extend funding for 2 more years (BMU 2021). In addition to funding for all municipalities, the federal government also supported climate policy in 41 forerunner municipalities, which were selected in two waves in 2012 and 2016 on a competitive basis and obtained initial funding for developing strategies to become climate neutral. The scheme is based on the assumption that these “Masterplan Municipalities” (*Masterplankommunen*, (*MPK*)) serve as “good practice” beacons for other municipalities.

**Fig. 2:**
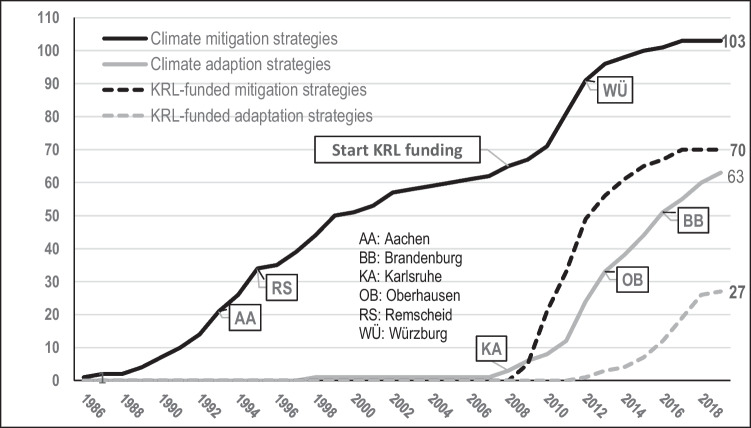
Federal funding of climate mitigation and climate adaptation strategies among the 104 largest German cities (KRL: Kommunalrichtlinie)

Figure 2 shows how federal funding underpinned the vertical upscaling of climate strategies in the 104 largest German cities from 2008 onwards. All of these cities took advantage of KRL funding and developed or updated their mitigation strategies, with the result that 103 of them had adopted plans by the end of 2018. Seventy of these initial or revised strategies were funded through the KRL, as were 27 of the 63 adaptation strategies (Fig. 2). In addition to the KRL programme, which also supported integrated mitigation and adaptation strategies alongside individual plans for each sector,^3^ the federal government recently started the programme “Measures for Climate Change Adaptation” (*Maßnahmen zur Anpassung an den Klimawandel*), which is part of the German Adaptation Strategy.^4^ This suggests that federal and state governments need to step in to ensure that all municipalities have enough resources to take action and latecomers will not be left further behind (Kern 2019). In addition, Fig. 1 illustrates that the introduction of the KRL even stimulated latecomers to join the Climate Alliance. Out of a group of 31 latecomers, which had not joined the Climate Alliance in the 1990s, twelve cities (including Würzburg) joined the network around the same time as setting up their climate strategies.

While the transfer of climate mitigation practices in the 1990s was triggered primarily by transnational municipal networks (Kern and Bulkeley 2009), the emergence of climate adaptation strategies during the last decade has been influenced by federal and state funding programmes from the outset (Häußler and Haupt 2021; Graf et al 2018). Moreover, vulnerability to increasing climate impacts is likely to act as an additional catalyst for developing adaptation strategies (Aguiar et al 2018; Kern et al 2021). Since there are more municipal networks for climate mitigation than for adaptation, (horizontal) diffusion seems to be less influential for adaptation than for mitigation strategies (see Fig. 2).

Federal and state funding programmes for developing climate strategies have changed the dynamics between climate policy forerunners, followers and latecomers by stimulating initiatives in municipalities that would otherwise have been less likely to respond. Funding also caused the convergence of local climate strategies due to the specific programme requirements and the emergence of consultancies, which developed templates and adjusted them to the context of particular cities.

Orchestrated by the federal government, we can see how the spread of climate strategies has become embedded in the German multilevel system. Funding programmes such as the KRL scheme led to new forms of vertical interaction between the federal government, the state governments and a growing number of municipalities (Graf et al 2018). However, horizontal diffusion led to forerunner cities developing climate strategies, hiring climate managers and cooperating with each other long before the federal government stepped in. As cities were not required to take action on the issue, setting up funding programmes such as the KRL and similar state programmes led to vertical upscaling.

With respect to the MPK programme for forerunners, which involved establishing a network of all MPK municipalities and facilitating knowledge exchange between them, we can see how horizontal diffusion amongst forerunner cities is transformed into horizontal upscaling when the federal government intervenes and seeks to direct climate policy in the participating cities. The rationale behind this federal initiative is the assumption that nationally funded beacon projects in forerunner cities will serve as a blueprint for other municipalities. However, horizontal diffusion may work only within but not beyond the privileged group of forerunners, because non-participating municipalities do not get the same attention and support. Thus, horizontal diffusion of such programmes is most often limited, as followers, and in particular latecomers, lack the resources to catch up. This shows the limits of horizontal diffusion amongst forerunners and the need for horizontal upscaling to reach the followers and latecomers.

### Pathways of climate mitigation and adaptation strategies in German cities

We now analyse the pathways of climate policy in forerunner, follower and latecomer cities for both mitigation and adaptation—based on the four dimensions of policy diffusion and upscaling. Although our focus is primarily on the factors that facilitate the spread of climate strategies, we also highlight other related initiatives within each city, to illustrate their varying levels of activity and ambition.

#### Mitigation forerunner: Aachen

Aachen joined the Climate Alliance in 1991 as one of the first cities in Germany and before the first diffusion wave between 1992 and 1995 (see Fig. 1). Through its decision to join the network, which needed to be underpinned by a city council resolution, Aachen was also one of the first German cities to set a climate mitigation goal. In 1993, the city released its first mitigation-related strategy. Over time, Aachen has passed several additional climate strategies and action plans including a CO_2_ reduction strategy (1998), an energy efficiency strategy (2006) and two mitigation strategies (2013, 2020).

Although the 2013 strategy was funded through the KRL programme, the long list of strategies and action plans adopted by the city council shows that Aachen’s climate activities never relied heavily on external support. Depending on the political majority in the city council, Aachen was sometimes more, sometimes less, active in climate policy (interview A1). Nevertheless, since the early 1990s, it has been consistently amongst the most ambitious cities in Germany (Otto et al 2021; Irmisch et al 2022). Triggered by the horizontal diffusion of climate emergency declarations amongst German cities in recent years, Aachen passed such a resolution in June 2019, pressured by a strong and active civil society (including Fridays for Future). In addition, Aachen elected a Green Party mayor in 2020. The climate emergency declaration and the change of political leadership were quickly followed by a new climate strategy and an action plan in 2020. It includes 70 specific mitigation and adaptation measures, which are funded by the city with around 80 million Euros (interview A1).

#### Mitigation Follower: Remscheid

Remscheid joined the Climate Alliance in 1995, after the initial forerunners (see Fig. 1). This marked the starting point of distinct and targeted climate action (Otto et al 2021). In the same year, Remscheid published its first climate report as well as a CO_2_ reduction plan (together with the neighbouring cities Solingen and Wuppertal). Just as in Aachen, the emission reduction goals associated with Climate Alliance membership were Remscheid’s first mitigation goals, illustrating the close connection between joining the network and developing initial plans. In 1999, Remscheid published its first mitigation strategy.

Joining the Climate Alliance was not without obstacles for the Environment Department, which took the lead on climate action within the city administration (interviews R1 and R2). Remscheid, a city chronically short of budgetary resources, was unable to pay the membership fee. Thus, staff from the Environment Department launched a fundraising campaign within the city government to cover the first annual fee (interview R1). Even today, resource constraints remain the main obstacle for local climate action in this shrinking city marked by industrial decline (interviews R1, R2 and R3). As a result, all major initiatives have been financed through either funding schemes or participation in third-party-funded research projects. For example, Remscheid’s first mitigation strategy (1999) and its participation in the certification programme “European Energy Award” (2003, 2007 and 2018) were funded by the state of North Rhine-Westphalia. Remscheid’s second mitigation strategy (2014), the temporary employment of a climate manager (2017–2019) and the development of a mobility strategy (2018) were funded through the KRL programme*.* Overall, funding provided by federal and state governments was crucial for Remscheid’s climate activities (vertical upscaling). The focus on third-party-funded climate policy can also be explained by relatively little political support from the city’s mayors (Social and Christian Democrats) (interviews R1 and R2) (Haupt and Kern 2022).

#### Mitigation latecomer: Würzburg

As in Aachen and Remscheid, entry to the Climate Alliance in 2008 marked the start of Würzburg’s first distinct and targeted climate actions. This was at the beginning of a second diffusion wave of entries into the network between 2007 and 2015 (Fig. 1). As in Remscheid, there were 4 years between joining the Climate Alliance and publishing the first climate strategy. Unlike most German cities of similar size, Würzburg became active in climate policy very late (Otto et al 2021; Kern et al 2021). Nevertheless, it pursued an ambitious climate policy after this delayed start. In 2012, the city presented an integrated mitigation and adaptation strategy. As Würzburg is located in a basin and has only a few green areas, it regularly experiences a strong urban heat island effect. Therefore, the city decided to tackle adaptation from the outset (interviews W1 and W3). The integrated strategy was funded through the KRL (vertical upscaling) and has been Würzburg’s central strategic climate policy instrument before the city adopted a revised and more ambitious integrated strategy in 2022, which aims for climate neutrality by 2045 and was funded by the state of Bavaria.

Nonetheless, as a prosperous university city with a comparatively young and well-educated population, Würzburg has the means to act independently on climate change. Horizontal diffusion is therefore as important as vertical upscaling. Moreover, Würzburg has demonstrated substantial efforts to integrate climate policy into local politics and administrative action (interviews W1, W3). In 2010, a climate coordination office was created, and in 2016 a climate advisory board was set up. After the local elections in 2020 when the Green Party achieved its best ever election result, the city established a department for environmental affairs and climate change and appointed a so-called climate mayor (a deputy mayor), the first of its kind in Bavaria. To conclude, after a delayed start, Würzburg is now on the way to catch up to the forerunners, based on favourable local conditions, driven by horizontal diffusion and facilitated by vertical upscaling.

#### Adaptation forerunner: Karlsruhe

Karlsruhe was one of the first German municipalities to take steps to address the impacts of climate change (Jolk 2015; Hogrewe-Fuchs  2017). Building on its first mitigation strategy from 1999, the city published a report in 2008, setting out how it was particularly vulnerable to heat stress, drought and invasive species, because the average number of days on which the temperature was predicted to exceed 30 °C by 2050 would be higher than in any other city in southern Germany. Karlsruhe’s position as a place that is highly vulnerable to climate impacts fed into its first adaptation strategy of 2013, which provides a comprehensive overview of how climate change will affect different sectors and sets out a detailed list of initiatives to reduce its impact. Reflecting how seriously the city took its vulnerability and its position as a forerunner, this strategy was wholly funded by the municipality (Jolk 2015). By this time, Karlsruhe had already developed a mitigation strategy and established a city energy and climate agency to advice residents and businesses (interview K1). Karlsruhe also published an updated adaptation strategy in 2021, confirming its place as a leading city (Otto et al 2021). As a wealthy university city with strong Green Party representation (Irmisch et al 2022), it also benefited from favourable local conditions for progressive climate policy.

Karlsruhe’s position as an adaptation forerunner meant there were few appropriate models to emulate and therefore horizontal diffusion was very limited. Staff across municipal departments had a high awareness of climate issues and the city’s particular vulnerability to heat stress (interview K2), which contributed towards the decision to come up with a strategy. Nonetheless, because managers recognised adaptation and mitigation as the two key pillars of Karlsruhe’s climate approach early on, they felt that each deserved similar levels of attention (Jolk 2015; Hogrewe-Fuchs  2017). Although Karlsruhe did not join the Climate Alliance until 2011, horizontal diffusion was an important driver for developing its mitigation strategy, which was largely copied from other cities. Many of the municipality’s adaptation initiatives are self-funded, but Karlsruhe was also able to access project funding from the state of Baden-Württemberg (Jolk 2015). Therefore, we can see how its approach was largely shaped by internal pressure for action and horizontal diffusion, albeit facilitated by a degree of vertical upscaling to fund certain measures.

#### Adaptation follower: Oberhausen

The example of Oberhausen illustrates the limitations of horizontal diffusion quite clearly, particularly where cities are experiencing financial constraints. Oberhausen is the second-most “sealed” city in Germany after Munich: non-permeable surfaces comprise 44% of the city’s territory, making it highly vulnerable to heat stress and pluvial flooding. The city joined the Climate Alliance in 1998 and has a long history of undertaking ad hoc mitigation and adaptation initiatives. However, GDP per capita is much lower than the German average (Table 1), the city’s population has shrunk since the early 1990s (Irmisch et al 2022) and it is one of Germany’s most indebted municipalities—to the extent that it operated within severe financial constraints between 1994 and 2016 (Schlick 2019). Thus, Oberhausen’s climate activities have all been financed by external project funding (interview O1) and undertaken on the basis that they would be cost-neutral for the city and help to improve its image and liveability (interview O2). Despite its early enthusiasm and vulnerability to climate threats, Oberhausen only developed a climate strategy after it received federal funding through the KRL in 2012. Although this strategy did include some adaptation initiatives, it focused more on mitigation. Furthermore, the city has also been able to access funding from the state of North Rhine-Westphalia, which provides funding for sixteen municipalities in the industrial Ruhr area to collaborate on climate resilience issues (*Zukunftsinitiative der Emschergenossenschaft*). As a result of this vertical upscaling, the city has been able to take its climate policy forward and expects to publish a stand-alone climate adaptation strategy in 2022 (interview O3).

#### Adaptation latecomer: Brandenburg an der Havel

The case of Brandenburg/Havel also shows how vertical upscaling through central funding schemes is often a crucial factor in developing local climate policy. Following a period in which the municipal budget was in deficit, between 2014 and 2018, it had to operate under strict financial restrictions and report to the state government on its progress in balancing revenues with expenditures. Even after these restrictions ended, the Christian Democratic mayor elected in 2019 still viewed reducing municipal debt as his overriding priority (Irmisch et al 2022). This led the city treasury to forbid spending on any non-statutory services or projects. Since addressing climate change remains a voluntary function in German local government, it had to bid for external funding to develop climate policies, even though climate change became an increasingly important issue within the city council after the Paris conference in 2015 (interview B1). As such, the city only developed a climate strategy in 2017 after it had successfully applied for KRL funding (vertical upscaling) (interview B1), but it never joined the Climate Alliance. In contrast to Oberhausen, there are far fewer opportunities than in other states to access funding from the state of Brandenburg (Eckersley et al 2021), which limits the opportunities for vertical upscaling. Indeed, the lack of more obvious funding schemes and the difficulties associated with calculating the costs and benefits of adaptation initiatives meant that the city included a chapter on adaptation within its climate protection strategy, rather than developing a specific climate adaptation strategy.

## Comparing diffusion and upscaling of climate mitigation and adaptation strategies

Comparing the spread of climate mitigation and adaptation strategies reveals similarities as well as differences between both pillars of climate policy. In both areas, we find a lack of hierarchical authority from higher levels of government since climate policy is still a voluntary task of German municipalities. Thus, the adoption of climate mitigation and adaptation strategies was primarily driven by horizontal diffusion and vertical upscaling. As we anticipated, cities that are more vulnerable to climate threats were more likely to introduce adaptation strategies, and local socioeconomic and political conditions also shaped the extent to which each city became engaged in horizontal networks (horizontal diffusion) and applied for state and federal funding (vertical upscaling).

Local climate mitigation strategies emerged almost 20 years earlier than climate adaptation strategies. From the early 1990s onwards, in many German cities, the spread of climate mitigation strategies was triggered by joining the Climate Alliance. Particularly between 1992 and 1998, we see Climate Alliance membership diffusing rapidly, and a concomitant trend in the spread of mitigation strategies. Between 1998 and 2008, membership of the Climate Alliance was relatively stable, although only about 60% of the 104 largest German cities had joined (Fig. 1). Membership increased again after 2008. Cities like Würzburg, which had been passive until then, started from scratch and thus found it attractive to join the Climate Alliance, which is still the most prominent transnational municipal network in Germany. We assume that this trend is associated with the launch of the KRL in 2008. Following its introduction, the adoption of climate mitigation strategies accelerated again (Fig. 2) and stimulated even latecomers to take action. Therefore, vertical upscaling changed the situation in almost 40% of the largest German cities, which introduced climate mitigation strategies only after the KRL programme had started in 2008 (Figs. 1 and 2). Without KRL funding, this increase would most likely not have taken place.

In contrast to climate mitigation, the spread of climate adaptation strategies was influenced by federal and state funding from the outset. As the first adaptation strategies emerged at almost the same time as the KRL programme was introduced, horizontal diffusion between municipalities was rather limited in this policy area. The initial development of adaptation strategies was driven by local vulnerability to climate impacts, but their spread was influenced more by vertical upscaling than by horizontal diffusion, because fewer networks for climate adaptation exist and federal and state funding was available from the outset.

However, size matters for both pillars of local climate action; i.e. bigger cities are more likely to start earlier than smaller ones, and they are also more likely to have sufficient capacities to become active in both areas. Smaller cities tend to be latecomers in both areas of climate policy due to a lack of capacities and their dependency on external resources. Generally speaking, smaller cities joined the Climate Alliance later than their bigger peers and they also introduced climate mitigation and adaptation strategies later. Although external financial support is important for all German municipalities, smaller and poorer municipalities depend far more on it. This means that horizontal diffusion has its limits because joining a transnational municipal network and institutionalising climate policy requires certain capacities.

Dependence on external support is also evident with respect to our case studies. Forerunner cities (Aachen, Karlsruhe) are less dependent on external funding, because they have more (financial) capacities, partly due to their more favourable political and socioeconomic conditions. Follower cities (Remscheid, Oberhausen) need models to follow, i.e. comparable cities which have already become active. Key actors in the city administration can then use these examples to convince local politicians that local climate action is needed. This means that horizontal diffusion is important for follower cities because they focus on their peers and learn from the experience of forerunners. However, our case studies also show that their performance improves considerably by vertical upscaling because they depend more on external funding than the forerunners. Finally, the climate policy pathways in latecomer cities such as Brandenburg/Havel suggest that vertical upscaling is most important for these municipalities, whereas horizontal diffusion is limited. The city became active only after federal funding was available, and we can assume that it would have remained passive without it. Although Würzburg undertook climate action much later than comparable cities, its more favourable local conditions mean that this city is in a much better position to catch up with the forerunners.

Overall, this means that the adoption of mitigation and adaptation strategies was influenced mainly by two factors: the foundation of the Climate Alliance in 1990 and the introduction of the KRL funding programme in 2008. While the foundation of the Climate Alliance by forerunner cities led to a rapid diffusion of membership and triggered horizontal diffusion, the introduction of the KRL programme shows the importance of vertical upscaling for the spread of climate policies. The differences between climate mitigation and adaptation strategies can be explained by the fact that some cities are much more vulnerable to climate impacts than others (Aguiar et al 2018; Kern et al 2021) and that the development of climate mitigation strategies started at a time when federal funding was not yet available.

Voluntary horizontal policy diffusion is most likely to occur amongst forerunners because they have the best preconditions to learn from experiences elsewhere. The climate policy pathways of followers are also characterised by horizontal diffusion, but vertical upscaling plays an equally important role. For latecomers, vertical upscaling is even more important because they do not have enough capacities to adopt policies developed by forerunner cities. Indeed, the combination of favourable local preconditions and vertical upscaling helps to bring slower cities up to the speed of the forerunners. The dynamics between forerunners, followers and latecomers has long been neglected in the analysis of local climate action because research has focussed primarily on forerunners. As forerunners have higher capacities to start and promote policy innovations, focusing on them leads to a blurred perspective, which overestimates the importance of horizontal policy diffusion, while underestimating the need for upscaling initiatives of higher levels of government.

Thus, we argue that federal and state funding is essential for local climate action. Our analysis revealed that the effects of horizontal diffusion are limited because a considerable number of municipalities will take action only if external funding is available. External funding is important for follower cities and even more essential for latecomers, which do not benefit from the same internal drivers as the forerunners. Moreover, in the case of climate adaptation strategies, vertical upscaling has been an important driver for their spread from the outset.

## Conclusions

It can be concluded that the spread of local mitigation and adaptation strategies across Germany can be best explained by a combination of horizontal policy diffusion and vertical upscaling, whereas vertical diffusion and horizontal upscaling have played only a limited role. By arguing that upscaling of local climate policies from forerunners to followers and latecomers depends on interventions by federal and state authorities, we stress that voluntary diffusion needs to be supplemented by upscaling initiatives from higher levels of government. Municipalities require advice and resources from elsewhere in order to develop and implement effective climate strategies. Institutional arrangements and the embeddedness of local climate policy in the German multilevel system therefore play a key role in shaping whether and how cities address the climate emergency.

We would welcome further research into these dynamics between forerunners, followers and latecomers, particularly in countries with different intergovernmental systems and different levels of local (financial) autonomy. We suggest that vertical upscaling may be particularly important in countries with a high number of smaller municipalities, such as France, Italy and Austria. Furthermore, although we focused largely on horizontal diffusion and vertical upscaling, studies that examine whether and how all four dimensions operate within different multilevel arrangements could also reveal important insights.

## Supplementary information

Below is the link to the electronic supplementary material.

Supplementary file1 (DOCX 13 KB)

## Footnotes

1 In this paper, we use the term state(s) for the 16 German federal states (*Länder*), and the term federal government for the national government.

2 All municipalities with over 100,000 residents, and all municipalities over 50,000 inhabitants that are not part of a county.

3 Integrated climate strategies are counted for both areas of climate policy.

4 The KRL programme has focused mainly on climate mitigation, but until 2018 municipalities could apply for funding of integrated climate strategies and climate adaptation strategies. In 2021, a new funding programme was launched, which provides funding for climate adaptation strategies and climate adaptation managers.
