# Supplementary material for: Diffusion and upscaling of municipal climate mitigation and adaptation strategies in Germany
Source: Reg Environ Change. 2023 Jan 20;23(1):28. doi: 10.1007/s10113-022-02020-z (PMC9853482; doi:10.1007/s10113-022-02020-z)
Supplement: Supplementary file 1 — Supplementary file1 (DOCX 13 KB) [file 10113_2022_2020_MOESM1_ESM.docx]

**Annex 1**

| **City** | **Interviewees’ affiliation** | **Dates of interviews** |
| --- | --- | --- |
| Aachen (AA) | Environment Department (1 interview) | November 2020 |
| Remscheid (RS) | Environment Department (3 interviews) | June 2019; September 2019 |
| Würzburg (WÜ) | Environment and Climate Department (3 interviews) | March 2018; January 2021 |
| Karlsruhe (KA) | Environment Department; Energy and Climate Agency (2 interviews in total) | July 2021; September 2021 |
| Oberhausen (OB) | City Development and Environment Department (3 interviews) | November 2020; February 2021 |
| Brandenburg an der Havel (BB) | City Development Department; City Council (Green Party); local mobility consultancy (3 interviews in total) | November 2021 |

*List of interviews*
